# Exploring the Potential of Atlantic Mesopelagic Species Processed on Board Commercial Fishing Vessels as a Source of Dietary Lipids

**DOI:** 10.3390/foods13071094

**Published:** 2024-04-02

**Authors:** Maria A. Madina, Eduardo Grimaldo, Leif Grimsmo, Bendik Toldnes, Rasa Slizyte, Ana Karina Carvajal, Marte Schei, Merethe Selnes, Eva Falch

**Affiliations:** 1 Department of Fisheries and New Biomarine Industry, SINTEF Ocean, 7010 Trondheim, Norway; 2 Department of Biotechnology and Food Science, NTNU—Norwegian University of Science and Technology, 7491 Trondheim, Norway; 3 The Norwegian College of Fishery Science, The Arctic University of Norway (UiT), 9037 Tromsø, Norway

**Keywords:** marine oils, omega-3, EPA, DHA, thermal separation, hydrolysis, enzymes, krill, *Maurolicus muelleri*, *Benthosema glaciale*

## Abstract

This study investigates the use of untapped mesopelagic species as a source of long-chain polyunsaturated omega-3 fatty acids (LC n-3 PUFAs) to meet the growing demand. The challenges faced by commercial fishing vessels, such as varying catch rates and species distribution affecting lipid levels, are addressed. Marine oils were produced post-catch using thermal separation and enzymatic hydrolysis during four commercial cruises, screening approximately 20,000 kg of mixed mesopelagic species. *Maurolicus muelleri* and *Benthosema glaciale* were the dominant species in the catch, while krill was the primary bycatch. The lipid composition varied, with *B. glaciale* having a higher prevalence of wax esters, while triacylglycerols and phospholipids were more predominant in the other species. LC n-3 PUFAs ranged from 19% to 44% of lipids, with an average EPA + DHA content of 202 mg/g of oil. Both processing methods achieved oil recoveries of over 90%. Estimates indicate that the mesopelagic biomass in the Northeast Atlantic could supply annual recommended levels of EPA + DHA to 1.5 million people, promoting healthy heart and brain functions. These findings offer valuable insights for considering mesopelagic species as a potential source of dietary marine lipids, laying the groundwork for further research and innovation in processing and obtaining valuable compounds from such species.

## 1. Introduction

With the expanding global population and increasing demand for food, there is a growing reliance on the ocean to meet the need for seafood. Deep-sea fishing has been proposed as a viable approach to support the growing food supply. The mesopelagic zone is believed to contain a substantial 1.8–16 billion metric tons of fish biomass, which constitutes approximately 50 to 90% of the total mass of fish on earth [1]. This area contains a variety of fish species, including bristlemouth fish and lanternfish, as well as other deep-sea creatures such as krill, crustaceans, squid, cephalopods, and gelatinous organisms like jellyfish [1,2].

Mesopelagic species can provide a rich source of nutrients that may significantly contribute to global food and feed production. The nutritional analysis of various mesopelagic species has revealed high levels of vitamin A1, calcium, selenium, iodine, and the LC n-3 PUFAs eicosapentaenoic acid (EPA) and docosahexaenoic acid (DHA) [2,3]. The nutritional profile, which includes valuable marine lipids and proteins, has been compared with that of other commercially relevant species [2]. Peruvian anchoveta (*Engraulis ringens*) is a major contributor to the global production of EPA and DHA [4]. Mesopelagic species caught in western Norway fjords contained up to 30% LC n-3 PUFAs [3], a level comparable to that found in Peruvian anchoveta [4]. The demand for marine oils has increased due to the growing recognition of the health benefits associated with LC n-3 PUFAs, which are primarily sourced from marine and aquaculture sources [5]. As the aquaculture industry continues to expand, mesopelagic species have been proposed to meet the demand for wild-harvested marine fatty acids.

While mesopelagic fish comprise a substantial portion of the global biomass, their densities in specific locations can be relatively low. This necessitates large-scale extraction efforts for economic viability. Despite the ambitious commercial interest in these species, commercial trial fisheries in the Northeast Atlantic experienced challenges such as large and unpredictable variations in mesopelagic catch rates, the presence of multiple species in the catch, and variable amounts of bycatch [2,3]. Off the coast of Norway, several trials targeted mesopelagic species in 2018, 2019, 2020, and 2021. The catch rates for these years were 31, 1693, 101, and 146 tons, respectively [6,7]. Most of the catch consisted of *M. muelleri*, and krill species dominated as bycatch, constituting an average presence of 18% [6]. Large fluctuations in the catch rates of mesopelagic species might compromise the economic feasibility of a potential fishery. The presence of multiple species in a catch also challenges the preservation, further processing, and potential use of the catch as a marine source of ingredients [2,8]. Previous studies have shown that variations in catch size correspond to variations in the nutritional profile, particularly regarding oil content and composition [2,3].

Furthermore, mesopelagic species degrade quickly after harvest if not handled and processed correctly. This is due to the activation of digestive enzymes, which causes rapid autolysis [2,9]. As a result, the raw material becomes highly perishable, leading to a degradation in quality and a loss of nutrients, even when stored under freezing conditions. On-board processing enables the rapid conversion of freshly caught marine species into value-added products rich in marine proteins, oils, and other derivatives [8,10]. This approach aims to maximize the freshness of the catch and reduce the time between harvest and processing. However, the need for specialized equipment, skilled personnel, and stringent hygiene standards adds complexity to on-board processing operations. On board fishing vessels, a frequently used method is the wet rendering process, which involves thermal processing of the raw material. First, the material is heated to 90–95 °C to deactivate autolytic enzymes. Subsequently, the material is cooked, minced, and heated to coagulate proteins and release water and oil. Finally, the cooked material is separated into three phases (a solid phase, a water phase, and an oil phase) [11]. The use of the enzymatic hydrolysis of by-products from fish and fisheries (i.e., viscera, heads, processing remains) is also a well-known technology for regaining oil and producing highly digestible and bioavailable protein products while preserving its functional properties [12].

Several studies have evaluated the biochemical composition of different mesopelagic species [2,3,13,14,15,16], but scientific literature is scarce on the production of marine-derived ingredients, particularly marine oils. The potential of these species has also been evaluated based on predicted biomass estimations, but it is documented that mesopelagic catch rates are highly variable [2,6,7]. Conducting studies of this nature is essential to gain a better understanding of the potential contribution that such species could make to food and nutrition security, representing a potential harvestable resource for future commercial exploitation, either through direct human consumption or indirectly as feed ingredients in aquaculture.

This study is the first to assess the potential of mesopelagic species for marine oil production. The research is based on nearly 20,000 kg of raw material harvested in the Atlantic Ocean over a four-year period, reflecting the practical feasibility of the commercial harvesting of such species. Marine oils were produced rapidly on board within hours after harvest using thermal separation and enzymatic hydrolysis, two of the most used methods for on-board processing. The presented results provide a comprehensive insight into Atlantic mesopelagic species for marine oil production and establish a foundation for future research. This provides valuable information for estimating the potential contribution of these species to meeting the increasing demand for LC n-3 PUFAs.

## 2. Materials and Methods

### 2.1. Raw Material

**Figure 1:**
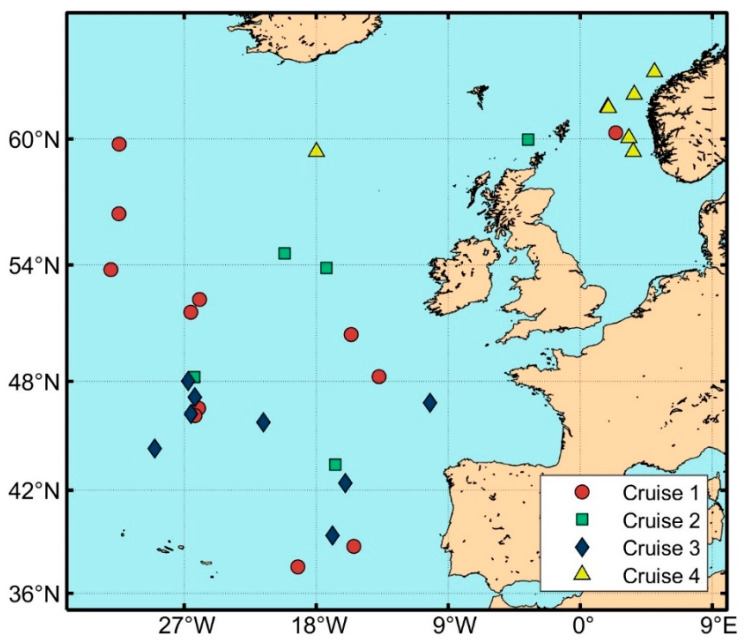
The map indicates the locations of the trawl hauls conducted during Cruises 1–4. The latitude on the *y*-axis and longitude on the *x*-axis indicate the geographical position of the hauls. Twelve hauls were conducted during Cruise 1 (shown in the map), but only samples from hauls 1–10 were used for the analysis and processing of the raw material (see Table 1).

Mesopelagic species were collected in trawl hauls on four separate cruises between 2016 and 2019 on board the 62 m long pelagic trawler “MS Birkeland”. Cruise 1 was conducted between 27 June and 29 July 2016; Cruise 2 between 18 April and 11 May 2017; Cruise 3 between 11 July and 4 August 2017; and Cruise 4 between 7 and 19 November 2019. Cruises 1, 2, and 3 took place in the Northeast Atlantic (Mid-Atlantic Ridge area), whereas Cruise 4 was conducted in the North Sea (Figure 1). A total of 29 trawl hauls were analyzed. Table 1 provides details about the trawl hauls’ depths and latitudes.

### 2.2. Sampling Procedure

Samples were collected using two pelagic trawls, one with a 1200 m and the other with an 800 m circumference in the mouth. A series of 40, 30, 20, and 16 mm small-meshed blinders were attached inside each trawl’s extension piece to avoid the escape of mesopelagic fish through net panels. The codends were blinded with a 16 mm mesh, and the mesh size of the rear-most part of the codend was 8 mm. Homogenized subsamples were taken as a representative catch of the unsorted mesopelagic species for further on-board processing experiments. We used unsorted subsamples because future mesopelagic fisheries most likely will collect a variety of different species.

### 2.3. Proximate Composition

Grimaldo et al. [2] previously published results for the proximate composition, lipid classes, and fatty acid composition for Cruises 1, 2, and 3. Samples from Cruise 4 were analyzed using the same procedures. These data were combined with the results from cruises 1, 2, and 3 to investigate the similarities and differences between all cruises. Proximate composition data from all cruises were used to supplement the results of the processing experiments.

### 2.4. Processing Technologies on Board Commercial Vessels

Thermal separation and enzymatic hydrolysis were selected as the processing technologies due to their established use in the industrial production of marine oils. The mixed catches of mesopelagic species leads to significant variations in the proximate composition from haul to haul, resulting in non-homogeneous samples. Therefore, using the two methods enables a broader approach to processing and testing their efficiency on the varying raw material. The experimental conditions were formulated using previous literature (the relevant studies are cited in the specific sections for thermal separation and enzymatic hydrolysis). The limitations of conducting research on board commercial vessels were considered in the experimental design. As the fishing vessels were devoid of analytical lab equipment, the researchers transported all necessary equipment onto the vessels for their experimental work. 

### 2.5. Thermal Separation

**Figure 2:**
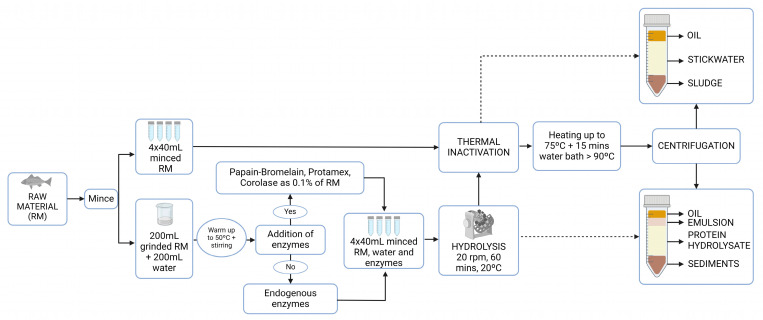
Flow chart showing the experimental design for the on-board processing experiments. In the flow chart, thermal inactivation refers to thermal separation. This method was used as a processing procedure, but it was also used to inactivate the enzymes during hydrolysis. Reprinted with permission from BioRender.com [2024].

During Cruises 1–4, thermal separation was conducted on board the vessel. Within 30 min of landing the catch, a 300 mL sample of representative raw material was minced using a manual grinding mill with an 8 mm hole diameter disk. For each station, four 50 mL graduated centrifuge tubes (replicates) were filled with 40 mL of minced fresh raw material. Thermal separation was carried out by heating the tubes in a microwave oven until the material reached ≈75 °C. The tubes were then placed in a boiling water bath for 15 min to ensure that the temperature reached >90 °C for inactivating endogenous enzymes [9,17]. After inactivation, the tubes were centrifuged in a rotary centrifuge (SL8 Centrifuge, Thermo Fisher Scientific, Waltham, MA, USA) at 2250× *g* for 10 min. The volumes of the yielded fractions of oil, stick water (water-soluble components), and sludge (insoluble components) were estimated by two independent persons who read the volume manually. Figure 2 shows the experimental design. The volumetric yield was then estimated as an average and standard deviation (SD) of the four replicates. The tubes containing the fractions were stored upright in a freezer at −20 °C and transported to the SINTEF’s SeaLab (Trondheim, Norway) for further analysis.

### 2.6. Enzymatic Hydrolysis

During Cruises 1 and 4, enzymatic hydrolysis processing was carried out on board the vessels. For all experiments, distilled water was mixed with 200 mL of minced raw material at a 1:1 ratio. This mixture was stirred and carefully heated in a water bath to a temperature of 50 °C [18]. Endogenous enzymes were not inactivated prior to enzymatic hydrolysis.

During Cruise 1, four different experiments were conducted to evaluate the effects of different enzymes on the hydrolysis process. The selection of these enzymes was based on prior experience and published findings [18]. Corolase PP (AB Enzymes GmbH, Darmstadt, Germany), Protamex (Novozymes A/S, Bagsvaerd, Denmark), and papain and bromelain (tested as a 50:50 mixture; Enzybel International, Waterloo, Belgium) were purchased from the respective manufacturers. The fourth treatment consisted of endogenous enzymes. During Cruise 4, Corolase PP was excluded from the experiments because it was no longer commercially available. The pre-weighed amount of commercial enzymes was added at 0.1% weight of the raw material. After stirring, 40 mL of the mixture (minced raw material, water, and enzymes) were transferred into four 50 mL graduated centrifuge tubes (replicates). The hydrolysis was performed by attaching the tubes to a rotor rotating at 20 rpm for 60 min at ambient temperature (approximately 20 °C). The temperature setting was limited by the constraints of operating on a fishing vessel. However, as the samples were preheated to 50 °C, this was the initial hydrolysis temperature. After hydrolysis, the samples were heated in a microwave oven until the mixture reached 90 °C, and the tubes were placed in a boiling water bath for 15 min to ensure the inactivation of all enzymes. The mixtures were centrifuged for 10 min at 2250× *g*, and then the volume of the yielded fractions [oil, emulsion, protein hydrolysate (water soluble components), and sediments (insoluble components)] were recorded as the average and SD of the four replicates (Figure 2). Considering the added amount of water, all values were divided by two to obtain results comparable to those from thermal separation. The tubes were stored upright in a freezer at −20 °C.

### 2.7. Statistical Analysis

The statistical software MINITAB v20 (https://www.minitab.com, accessed on 20 September 2023) was used for data processing and statistical analysis. The data were tested for normality and homogeneity of variances using the Shapiro–Wilk test (n < 50) and Levene’s F-test, respectively. When these parameters were confirmed, the variance was checked using one-way ANOVA. When samples were non-normally distributed, nonparametric Kruskal–Wallis tests were carried out. The means were compared according to post hoc comparisons, and significant differences were set according to Tukey’s honest significant differences test at the 95% confidence level (*p* < 0.05). The results are presented as mean values ± standard deviation (SD) for the proximate composition and mass distribution of lipids. The minimum and maximum values are given in brackets for the lipid classes, fatty acid composition, and thermal and enzymatic hydrolysis processing results. This was chosen due to the large variability found between most samples. Principal component analysis (PCA) was performed to investigate relationships within the lipid classes, fatty acid data set, and species contribution data.

## 3. Results and Discussion

### 3.1. Proximate Composition of Mixed Catches of Mesopelagic Species

The differences in catch composition between hauls and cruises resulted in a variation in the biochemical composition of mixed mesopelagic catches (Table 2). The proximate composition varied on different cruises, with the lipid content fluctuating the most, changing by a factor of 4.3 from the lower to the higher levels. The water content consequently varied, since lipids primarily constituted dry matter. By contrast, the protein and ash content remained relatively consistent.

The differences in lipid content may be due to the proportion of mesopelagic fish species compared to bycatch species, such as krill and jellyfish. Grimaldo et al., (2020) [2] observed that hauls with 80% fish generally had higher lipid contents compared to mixed hauls with significant bycatch. Our findings partially support this observation, as we recorded higher lipid contents during Cruise 1 and Cruise 4, where mesopelagic fish represented 71.5% and 69.2% of the catch, respectively. However, during Cruise 3, mesopelagic fish dominated the catch, comprising an average of 72.3%. Surprisingly, the lipid content was only half that of Cruises 1 and 4. No clear correlation was found when comparing the average percentage of fish with the average lipid content across different hauls and cruises. For example, one haul contained 100% of the fish *M. muelleri*, yet the total lipid content was only 1.9%. Similarly, hauls with 23.8% and 44.4% of fish contributed 12.2% and 12.4% of total lipids, respectively.

Based on this perspective, a higher incidence of bycatch may not always impede acquiring marine lipids. Blue whiting (*Micromesistius poutassou*), a pelagic fish species caught in the North Atlantic Ocean, is used for human consumption and is a significant European resource for fish oil and fishmeal production. Blue whiting typically contains 2–5% of lipids [2,19], which is comparable to the lowest lipid content observed in the mixed mesopelagic catches from Cruises 2 and 3.

These results highlight that the acquisition of marine lipids is influenced by various factors, including the diversity of species in the catch, as well as other biotic and abiotic factors that are not yet fully understood. For instance, in the Northeast Atlantic, krill is often the main by-catch species when mesopelagic species are harvested [6], and its lipid content can vary greatly depending on the season, location, maturity stage, and food availability [20]. The study’s findings have reinforced the long-standing debate about whether mesopelagic species should be harvested and exploited without a full understanding of the ecological and biological factors that influence mesopelagic biomass. Additionally, these results have raised intriguing questions that require further investigation, such as the lipid storage methods of various mesopelagic species in response to seasonal and latitudinal variations.

### 3.2. Lipid Classes and Fatty Acid Composition

An overview of the percentage of total lipids and the lipid classes is presented in Table 3, which shows that triacylglycerols, phospholipids, and wax esters were abundant in the catches collected during the different cruises. The fatty acid compositions presented in Table 4 show a particularly high content of DHA in all catches. The ratio between EPA and DHA was on average 3:7, which suggests that the mesopelagic species contained as much DHA as the reference values used for marine oil production [21].

Lipid classes varied throughout all cruises, primarily influenced by the association between individual species and specific lipid classes. This association was particularly evident when *B. glaciale* and *M. muelleri* dominated the catches, resulting in a notable increase in wax ester and triacylglycerol levels, reaching up to 85.4% and 89.9%, respectively. Most marine oils available commercially are present as triacylglycerols, ethyl esters, or phospholipids. Although the digestion and absorption mechanisms of triacylglycerols and phospholipids have been extensively studied, wax esters have generally been considered to be poorly digested in humans [1]. The consumption of fish rich in wax esters, when consumed in large quantities, has been reported to cause malabsorption with outbreaks of diarrhea, accompanied by stomach cramps, nausea, and vomiting. According to some publications, humans can digest wax esters in moderate amounts [22]. However, studies comparing the absorption of EPA and/or DHA from different lipid sources in humans have shown inconsistent results due to uneven fatty acid contents between the products tested [22]. Consequently, it may be necessary to decrease the EPA and DHA contents in wax ester-rich oils to enhance their absorption.

**Figure 3:**
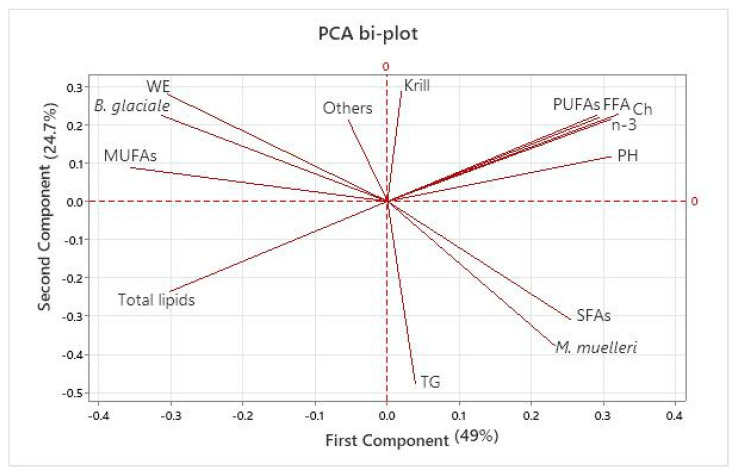
Principal component analysis (PCA) of the total lipid content, lipid classes, fatty acid profiles, and presence of mesopelagic species in the hauls. Eigenvalues in brackets represent the variance percentage explained by each axis (PC1 and PC2). The abbreviations for the lipid classes are as follows: WE: wax esters; FFA: free fatty acids; TG: triacylglycerols; Ch: cholesterol; PH: phospholipids. For the fatty acid profiles, the total sum of saturated fatty acids (SFAs), monounsaturated fatty acids (MUFAs), polyunsaturated fatty acids (PUFAs), and total sum of LC n-3 PUFAs were included in the PCA analysis. The term “others” refers to the presence of species other than *M. muelleri*, *B. glaciale*, or krill in the catch. *M. norvegica* likely dominated among krill species, but taxonomic identification was not performed and all of the krill species that came aboard as bycatch are included here.

Among all examined fatty acids, LC n-3 PUFAs were notably high, with an average EPA to DHA ratio of 3:7. However, the LC n-3 PUFA content was inversely related to higher lipid deposits (Figure 3). This tendency has been reported in previous studies, and a possible explanation is that when processing leaner catches, more of the omega-3 LC-PUFAs come from membrane phospholipids than from lipid deposits [13]. The association between phospholipids and the presence of LC n-3 PUFAs can be observed in the PCA plot (Figure 3), in agreement with previous research findings [2]. Consequently, it can be inferred that leaner raw materials, which are high in phospholipids, can provide a greater quantity of LC n-3 PUFAs.

Other abundant fatty acids were C16:0 (palmitic acid) and C18:1 n-9 (oleic acid). Palmitic fatty acids contributed on average 67% of the total saturated fatty acids (SFAs), while oleic acid accounted for 49% of the total monounsaturated fatty acids (MUFAs). The triad of palmitic acid, oleic acid, and DHA has also been found to be the most abundant group of fatty acids in marine oils from other fish species, including Baltic herring (*Clupea harengus*) [23] and Atlantic mackerel (*Scomber scombrus*) [24]. Associations were also identified between specific species and fatty acid groups. The PCA plot in Figure 3 illustrates that an elevated content of monounsaturated fatty acids (MUFAs) was observed when the abundance of *B. glaciale* was higher. By contrast, saturated fatty acid (SFA) contents were higher when *M. muelleri* was more abundant in the catches.

Overall, the variation in DHA content (14.7–22.4%) was higher than that for EPA (7.1–9.8%). This tendency can differ depending on the species. Baltic herring and Atlantic mackerel follow the same pattern observed for mixed catches of mesopelagic species [23,24], whereas Peruvian anchoveta (*Engraulis ringens*) has a higher content of EPA than DHA. The oil produced from Peruvian anchoveta accounts for 20% of the global fish meal and fish oil production, and its summed EPA and DHA content can range from 29 to 33% [4]. When comparing summed values of EPA and DHA, they ranged from 21.8 to 32.2% for the mesopelagic species. The contribution of these fatty acids from mesopelagic species is very relevant when considering Peruvian anchoveta as a reference for the global production of EPA and DHA [25].

Our results indicate that mesopelagic species could provide valuable marine oils based on their proximate composition, lipid classes, and fatty acid composition. These species comprise diverse lipid classes with varying rates of digestion and absorption, and understanding this could lead to the comparison of the absorption of essential fatty acids in different lipid forms and to the assessment of the potential suitability of oils derived from mesopelagic species for human consumption.

### 3.3. Thermal and Enzymatic Processing of Mesopelagic Species

Thermal separation and enzymatic hydrolysis are well-established methods for oil production. Thermal separation produced fractions of oil, stick water (containing water-soluble components), and sludge (containing insoluble components). Additionally, enzymatic hydrolysis resulted in four phases: oil, emulsion, soluble protein hydrolysate, and sediment (containing insoluble components). The yields of these fractions are presented in Table 5.

Both processing methods achieved oil recoveries exceeding 90%, demonstrating their effectiveness in generating high oil outputs. However, the oil recovery rates varied widely, ranging from 27% to 96%. No statistical differences were found in the oil yield between the two methods used in this study. However, on average, thermal separation resulted in a 15% lower oil recovery compared to enzymatic hydrolysis. Variations in oil recovery rates may be attributed to differences in the catch composition and the subsequent proximate composition. The lipid content of the raw material showed a positive correlation with the oil yield, indicating that a higher lipid content led to greater oil yields. This reinforces the relationship between the biochemical composition of the raw material and the subsequent oil yield. Likewise, the lipid composition can greatly influence the performance of processing technologies and the resulting yield and quality of the product. Although commercial pelagic fish are generally rich in triacylglycerols, our research has shown that the lipid classes present in mesopelagic species vary widely, with triacylglycerols, wax esters, and phospholipids often dominating [2,3,13]. Phospholipids are polar lipids that are mainly present in cell membranes, and they are quite abundant in krill species [20]. However, their polar configuration prevents them from entering the oil phase during thermal separation, and thus a high phospholipid content results in lower oil fraction yields when following this method. Triacylglycerols and wax esters are classified as neutral lipids and can enter the oil phase, increasing the oil yield during separation [26].

The relationship between lipid classes and the oil recovery rates can be inferred from Table 5. Cruise 1 and 4 produced the highest oil yields, and oil recovery rates ranged from 60% to 97% and 67% to 92% respectively. Cruise 1 had a combined 85% presence of triacylglycerols and wax esters, which easily enter the oil phase. No data are available for the lipid classes from Cruise 4, but the presence of *M. muelleri* fish was dominant at 70%, and it is likely to contain high levels of triacylglycerols, as suggested by the PCA plot. As previously discussed, a catch composition that is diverse may still contain a significant lipid contents. However, further processing for oil production may be difficult because of this premise.

The selection of processing technologies should be based on the characteristics of the raw materials and the resulting products.

The efficiency of processing technologies can also be evaluated based on the sediments generated: less sediment would indicate the better solubilization of proteins, better lipid separation, and fewer insoluble constituents, and thus a greater efficiency of the processing method [27]. In Cruise 1, hydrolysis with endogenous enzymes resulted in a higher sediment yield and a lower protein hydrolysate yield compared to both thermal separation and enzymatic hydrolysis with commercial enzymes (*p* < 0.05). A similar trend was observed in Cruise 4, although no significant differences among processing methods were found. This could be attributed to the specificity of enzymes. While commercial enzymes (proteases) are limited to protein hydrolysis, endogenous enzymes, consisting of a variety of enzymes, can catalyze different components beyond proteins. Thermal separation would not catalyze but modify diverse molecules through cross-linking reactions that may end up in the sludge fraction [11]. The formation of emulsions also complicates and reduces the oil separation and the solubilization of proteins, resulting in a lower yield of pure fractions [12]. An emulsion is a mixture of two or more immiscible liquids, where one liquid is dispersed within the other. This happened during Cruise 1 for all enzymatic treatments, where emulsions represented between 7.9% and 17.9% of the mass balance. Commercial enzymes particularly gave 2 to 2.5 times larger emulsion fractions than endogenous enzymes. The absence of an emulsion phase when samples were treated by thermal separation may be due to the smaller amount of added water, and a milder proteolysis compared to longer enzymatic hydrolysis processes that can result in shorter peptides with emulsifying properties. Incorporating water and mixing during the process of enzymatic hydrolysis would also introduce extra air and create favorable conditions for the formation of emulsions [12,26].

The selection of processing technologies for optimal oil separation should be based on an understanding of the composition of the raw material. What is effective for oil extraction may not be the best for protein solubilization. Investigating the biochemical composition of the different fractions will help to understand the distribution of diverse components that could be further utilized. This knowledge is essential for increasing the utilization of mesopelagic species by diversifying their by-products.

### 3.4. Free Fatty Acids as Quality Indicators

Free fatty acids are liberated due to the presence of endogenous lipases in the raw material and their measurement is a well-established parameter by the industry for quality assurance. The presence of free fatty acids in oil samples is categorized as an important quality parameter for food and feed applications, with lower contents indicating a better quality of the oil. According to the limits set by the guideline specifications for crude fish oils [28,29], the free fatty acid content should not be >5%. For refined fish oils, the free fatty acid content should not exceed 1.5%. Based on these specifications, only the oil obtained from Cruise 1 would comply with the free fatty acids threshold in crude oils, while both Cruise 2 and Cruise 3 exceed this threshold by 2.6 and 5.6 times, respectively (Table 4). This would require further refinement, such as an alkali treatment [28].

A significant amount of free fatty acids can act as a substrate for further lipid oxidation, leading to oil degradation. In addition, the presence of different lipid classes can affect the formation of free fatty acids, as the breakdown of triglycerides and phospholipids is faster than that of wax esters. The PCA plot shown in Figure 3 reinforces this notion by showing a strong correlation between phospholipids and free fatty acid formation. The highest levels of free fatty acids were observed in Cruises 2 and 3. During these cruises, phospholipid levels were significantly elevated compared to Cruise 1 (*p* < 0.05).

Free fatty acids are liberated due to the presence of endogenous lipases in the raw material, but optimal handling procedures can prevent enzymatic activity to some extent [30]. Additional research would be necessary to evaluate how the composition of the catch affects the presence and formation of free fatty acids, and to determine whether optimal handling procedures could prevent their formation. Otherwise, those batches with a high free fatty acid content would require further refining processes to meet food and feed application thresholds, resulting in additional costs for the final oil product.

### 3.5. Mass Distribution of Lipids and LC n-3 PUFAs

The data presented in this work were based on almost 20,000 kg of mesopelagic species biomass. The largest catch rates contributed with 4000 kg of biomass, 632 kg of oil, and 122 kg of LC n-3 PUFAs (Table 6).

Mesopelagic species have the potential to provide significant amounts of valuable marine nutrients. Projections suggest that they could contribute 242 tons by 2030, rising to 1210 tons by 2040. These species could produce 16,214 kg and 81,070 kg of marine lipids and 4840 kg and 24,200 kg of LC n-3 PUFAs by the same years, based on these projections and the average lipid and LC n-3 PUFA contents from Cruises 1–4. The largest reported catch in a single haul when targeting mesopelagic species in Norway was 102,225 kg, according to data from LieGruppen AS (Straume, Norway) fisheries in 2019. Based on the previous averages, this would be equivalent to 6849 kg of lipids and 2044.5 kg of LC n-3 PUFAs. Approximately 20 million tons of raw materials are used annually to produce fishmeal and fish oil. There has been an increase in global fish oil production from marine residues, reaching 48% [25]. This is mainly driven by the scarcity of raw material volumes from key species, particularly small pelagics, which remains a major constraint. The total allowable catch (TAC) in 2024 for cod in the Northeast Arctic is 474,427 tons, of which Norway’s share is 212,124 tons [31]. Approximately 1000 kg of marine oils and 300 kg of omega-3 fatty acids could be obtained from the cod residues of an average daily catch [32]. If mesopelagic catches could consistently match those reported for 2019, they could be expected to contribute a significant competitive share. However, the uneven abundance and distribution of mesopelagic species in the Northeast Atlantic, coupled with their landing price and operational uncertainties, pose challenges compared to other marine resources currently in use, such as marine discards.

### 3.6. Dietary Lipids from the Mesopelagic Biomass

Expert bodies from various countries have issued recommendations for the optimal intake of EPA + DHA nutrients, usually ranging from 250 to 1000 mg/day [33]. Based on 500 mg EPA + DHA as a reference value, Hamilton et al. [34] reported that the current supply of EPA + DHA from seafood and fish oil for human consumption is 420 k tons/year. According to their study [34], the daily per capita intake of EPA + DHA is 149 mg, which only meets 30% of the global demand. In the present work, in order to investigate the potential supply of dietary lipids from the mesopelagic biomass, we considered the recommendations established by the European Food Safety Authority (EFSA) in 2010 [35], where the EPA + DHA daily adequate intake should be 250 mg to, e.g., support a normal function of the heart and brain [36]. Our study showed that the EPA + DHA levels differed across the four cruises (*p* < 0.05, Table 4), ranging from 80 mg to 293 mg, with an average content of 202 mg/g of oil (%).

**Figure 4:**
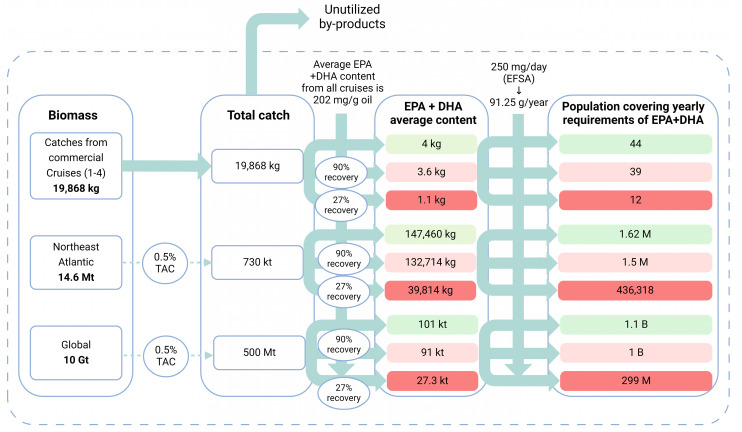
Graphical illustration of the provision of the dietary lipids EPA + DHA to the population from the mesopelagic biomass observed during Cruises 1–4, together with estimates of biomass in the Northeast Atlantic and globally. The units of measurement are represented by the letters M, G, and k, which correspond to million, giga, and kilo, in that order. Consequently, Mt, Gt, and kt refer to million tons, giga tons, and kilo tons, respectively. The arrows reflect the conversion from the biomass into an average content of EPA + DHA and the size of the population that would meet the yearly recommendations of 250 mg EPA + DHA. The dashed rectangles in green, pink, and red represent these outcomes under three different scenarios, according to oil recovery rates set at 27%, 90%, and 100%, respectively. These assumptions were made based on the lowest and highest oil recovery rates reported in Table 5, and a full recovery scenario. This means that the oil obtained from the biomass would represent 27% of the total lipid content, 90%, or the same amount as in the raw material. Based on this premise, an average content of 202 mg/g of oil (derived from Cruises 1–4) was used to calculate the average EPA + DHA provided by the three different biomasses and oil recovery rates. Reprinted with permission from BioRender.com [2024].

We calculated the population size that would meet the yearly adequate intake of 250 mg EPA + DHA provided by the mesopelagic biomass (see Figure 4). The extent of the mesopelagic biomass was assessed at three different scales using data from Cruises 1–4, assuming a total biomass of 14.6 million tons in the Northeast Atlantic [7] and a global estimated biomass of 10 Gt as predicted by Irigoien et al. [37]. In addition, we considered three different oil recovery rates when estimating the amounts of EPA + DHA derived from the oil: the lowest (27%) and highest (90%) recovery rates reported in Table 5, as well as a full recovery rate. Given the absence of a management plan for harvesting mesopelagic species, using a total allowable catch (TAC) of 0.5% was decided, mirroring the management plan for the copepod *Calanus* as a reference. Under this framework, a TAC of 0.5% of the estimated total biomass is deemed low and falls within safe biological limits [6].

Based on our findings, the supply of dietary EPA + DHA predicted from the Northeast Atlantic mesopelagic biomass could meet the yearly adequate intake [38] of 1.5 million people (representing 28% of the population in Norway). Additionally, the estimated global mesopelagic biomass has the potential to provide EPA + DHA benefits to approximately 1 billion people, which is equivalent to 12.5% of the global population. Furthermore, there is significant potential for utilizing the remaining by-products that are not intended for dietary oil production. These can be repurposed to produce bioactive peptides for human consumption [14], or feed for the expanding aquaculture sector [3]. Increasing the oil recovery from 27% to 90% could yield an additional 70% of EPA + DHA, highlighting the importance of developing optimal processing technologies. The development of such technologies has also been identified as a priority in our research, as the freshness of the raw material is critical to obtaining high-value by-products such as dietary lipids [8,10].

Our research has demonstrated the lipid-rich nature of mesopelagic species, suggesting a substantial potential contribution to the global EPA + DHA supply. Nonetheless, gaps persist in understanding the species- and stock-specific abundance and distribution. In addition, the projected biomasses exceed the reported catch rates from past trial fisheries. Moreover, significant uncertainties surround the overall environmental impacts of mesopelagic harvesting. Given these limitations, the remaining question is whether mesopelagic species can provide a significant contribution of EPA + DHA to meet current and future nutritional demands.

## 4. Conclusions and Future Perspectives

The assessment of almost 20,000 kg of mixed mesopelagic biomass provides evidence for the potential of these species in the supply of marine oil. Although the biochemical composition of mesopelagic species varies considerably from haul to haul, their contribution to LC n-3 PUFAs remains significant with an average EPA + DHA content of 202 mg/g oil. Our results also indicate that the diversity of lipid classes serves as a ‘trigger’ in determining whether lipids are separated into oil or not, with phospholipids being the main drivers of this process. Procedures for preventing the formation of free fatty acids should be developed to avoid the need for costly refinement processes. While further studies are needed to optimize processing conditions to control the oil separation and increase yield, our data provide evidence that it is possible to achieve oil recovery rates above 90% using both thermal separation and enzymatic hydrolysis. The characterization of the different fractions would also allow the upcycling of components other than oils to increase the use and value of the mesopelagic biomass. Based on estimates, the mesopelagic biomass in the Northeast Atlantic could supply the yearly adequate intake of EPA + DHA for 1.5 million people. Achieving these opportunities, though, depends on the proper management and processing of the mesopelagic biomass. The main question that remains, however, is how dense and widespread the mesopelagic biomass is in the Northeast Atlantic, and whether substantial and stable catches can be achieved in the future.

## Figures and Tables

**Table 1:** Trawl haul information, including date (DD MM YYYY), trawling start time (UTC), position (latitude and longitude), and maximum fishing depths (m) sampled. The same hauls were used for the biochemical analysis of the raw material and further processing trials.

Cruise No.	Station	Date (DD MM YYYY)	Time (UTC)	Trawling Time (min)	Position	Fishing Depth (m)
Cruise 1	1	28 June 2016	21:00	30	60°30′ N 04°37′ W	220
	2	2 July 2016	18:16	60	59°46′ N 31°26′ W	480
	3	3 July 2016	18:09	50	56°30′ N 31°27′ W	400
	4	4 July 2016	20:55	40	53°46′ N 32°00′ W	400
	5	5 July 2016	13:00	30	52°16′ N 25°57′ W	400
	6	5 July 2016	20:15	15	51°37′ N 26°33′ W	160
	7	7 July 2016	08:30	30	46°33′ N 26°01′ W	150
	8	7 July 2016	13:15	30	46°09′ N 26°16′ W	500
	9	13 July 2016	17:55	20	37°34′ N 19°15′ W	470
	10	14 July 2016	17:00	15	38°46′ N 15°26′ W	250
Cruise 2	1	21 April 2017	17:52	20	53°51′ N 17°19 W	100
	3	30 April 2017	18:00	60	48°13′ N 26°19′ W	320
	4	5 May 2017	11:53	45	43°27′ N 26°41′ W	50
	5	9 May 2017	17:52	45	55°34′ N 20°09′ W	199
Cruise 3	1	16 July 2017	10:10	130	47°10′ N 26°17′ W	260
	2	16 July 2017	18:47	45	46°15′ N 26°33′ W	212
	3	17 July 2017	14:30	50	44°20′ N 29°01′ W	338
	4	18 July 2017	18:53	50	48°00′ N 26°43′ W	212
	5	19 July 2017	20:09	60	45°47′ N 21°36′ W	85
	6	21 July 2017	16:25	50	39°24′ N 16°54′ W	212
	7	23 July 2017	15:27	60	42°24′ N 16°02′ W	170
	8	29 July 2017	13:20	30	46°52′ N 10°14′ W	100
Cruise 4	1	7 November 2019	11:45	50	59°25′ N 17°59′ W	180
	2	11 November 2019	10:45	60	59°25′ N 17°59′ W	245
	3	13 November 2019	09:30	122	59°25′ N 17°59′ W	269
	4	13 November 2019	13:40	60	59°25′ N 17°59′ W	266
	5	14 November 2019	10:12	73	59°25′ N 17°59′ W	252
	6	18 November 2019	11:52	61	59°25′ N 17°59′ W	241
	7	19 November 2019	10:32	67	59°25′ N 17°59′ W	244

**Table 2:** Biochemical and species composition of the raw material from the different cruises. The values were calculated as the percentage of wet raw material (g/100 g), and they are presented as an average of calculations with ±SD. Species composition is presented as the mean percentage of defined species in the catch.

Cruise	Lipid	Proteins (N × 6.25)	Ash	Moisture	*B. glaciale*	*M. muelleri*	Krill	Others
Cruise 1	9.9 ± 3.9	14.6 ± 1.2	2.9 ± 0.2	72.4 ± 3.0	41	42.6	10	5.9
Cruise 2	2.3 ± 0.7	14.5 ± 2.8	3.2 ± 0.4	79.8 ± 2.7	31	54.2	34	9.2
Cruise 3	4.9 ± 2.6	15.3 ± 1.5	3.3 ± 0.3	76.4 ± 2.8	7.8	64.5	25	2.6
Cruise 4	9.8 ± 2.4	11.8 ± 1.3	3.0 ± 0.1	75.7 ± 2.3		69.3	9	21.8

**Table 3:** Lipid content (% of total lipids) and lipid classes (% of total lipids) for Cruises 1, 2, 3, and 4 (C1, C2, C3, and C4, respectively). The results are presented as mean values, and the minimum and maximum values are given in brackets. Significant differences are presented between the cruises at the 95% confidence level (*p* < 0.05). The symbols “<” and “>” indicate if the value on the left is significantly higher or lower than the value on the right, respectively.

		Cruise 1	Cruise 2	Cruise 3	Cruise 4	*p* < 0.05
Lipid content (%)		9.9 (4.3–15.8)	2.3 (1.4–3.0)	4.9 (2.2–9.5)	9.8 (5.6–12.4)	
Lipid classes (% total lipids)	Wax esters	39.5 (0–85.4)	8.3 (0.6–29.4)	10.0 (0–40.2)	-	-
	Triacylglycerols	45.6 (3.3–89.9)	23.9 (5.0–54.4)	44.3 (11.3–77.5)	-	-
	Free fatty acids	1.8 (0.5–4.4)	27.9 (21.1–25.9)	13.3 (3.1–25.3)	-	C2 > C3 > C1
	Cholesterol	1.6 (0.5–4.3)	11.1 (6.3–16.2)	6.8 (2.6–11.4)	-	C2 > C3 > C1
	Phospholipids	16.5 (8.9–38.3)	28.8 (17.1–41.1)	24.5 (15.6–29.5)	-	C2 > C1

**Table 4:** Fatty acid composition (% of total fatty acids) as mean values from total numbers of hauls from Cruises 1,2,3, and 4 (C1, C2, C3, and C4, respectively) were used to explain analysis of variance among the different cruises (*p* < 0.05). The symbols “<” and “>” indicate if the value on the left is significantly higher or lower than the value on the right, respectively. The results are presented as mean values, and the minimum and maximum values are given in brackets.

Fatty Acid	Cruise 1	Cruise 2	Cruise 3	Cruise 4	*p* < 0.05
C14:0	5.0 (2.9–7.2)	5.2 (3.3–6.7)	4.7 (2.4–8.9)	6.9 (4.8–7.8)	-
C14:1	0.2 (0–0.3)	0.2 (0.1–0.3)	0.3 (0.3–0.4)	0.4 (0.3–0.4)	C1 < C3; C1, C2 < C4
C15:0	0.5 (0.2–0.8)	0.5 (0.4–0.6)	0.8 (0.5–1.1)	0.8 (0.8–0.8)	C1 < C3, C4
C16:0	15.5 (6.8–29.4)	19.6 (16.6–22.4)	25.3 (14.9–37.2)	21.4 (18.9–26.8)	-
C16:1 n7 + n9	6.5 (3.1–10.5)	4.8 (3.5–6.2)	3.8 (2.3–6.4)	4.4 (3.9–4.7)	C1 > C3
C17:0	0.5 (0.2–0.7)	0.7 (0.5–1.0)	0.8 (0.6–0.9)	0.6 (0.4–0.8)	C1 < C3
C17:1	0.5 (0.4–0.7)	0.7 (0.5–1.0)	0.7 (0.6–1.0)	0.5 (0.4–0.8)	C1 < C3
C18:0	2.5 (1.5–4.9)	3.1 (1.7–4.4)	3.9 (2.8–5.6)	2.3 (2.0–2.8)	C1, C4 < C3
C18:1 n11 + n9	18.5 (8.3–25.8)	13.1 (7.9–24.9)	16.8 (12.7–22.1)	11.5 (9.8–16.4)	-
C18:1 n7	1.9 (1.3–2.5)	2.7 (2.3–3.0)	1.4 (0.5–2.5)	1.7 (1.3–2.2)	C1, C3, C4 < C2
C18:2 n6	1.5 (1.2–1.9)	1.6 (1.2–2.0)	1.6 (0.9–1.9)	1.3 (1.2–1.5)	-
C18:3 n6	0.2 (0.1–0.2)	0.1 (0.1–0.2)	0.2 (0.1–0.3)	0.2 (0.2–0.3)	-
C18:3 n3	0.8 (0–1.4)	0.8 (0.5–1.2)	0.8 (0.6–1.1)	1.2 (0.9–1.5)	-
c18:4 n3	2.3 (0.5–3.8)	1.7 (0.7–3.5)	2.2 (1.5–2.9)	3.7 (2.8–4.6)	C1, C2, C3 < C4
C20:0	0.2 (0.1–0.3)	0.1 (0.1–0.1)	0.2 (0.1–0.3)	0.2 (0.2–0.2)	CC1, C3, C4 > C2
C20:1 n11+ n9 +n7	7.7 (1.8–11.8)	3.8 (1.7–9.3)	1.2 (0.4–2.0)	6.1 (1.6–8.1)	C1, C4 > C3
C20:2 n6	0.3 (0.2–0.4)	0.2 (0.2–0.3)	0.3 (0.4–0.4)	0.2 (0.2–0.3)	-
C20:3 n6	0.1 (0–0.2)	0.1 (0.1–0.2)	0.1 (0–0.1)	0.1 (0.1–0.1)	-
C20:4 n6	0.2 (0.1–0.3)	0.7 (0.5–0.8)	0.4 (0.2–0.7)	0.1 (0–0.1)	C1, C4 < C2 < C3
C20:3 n3	0.1 (0–0.1)	0.2 (0.1–0.3)	0.2 (0.1–0.3)	0.2 (0.1–0.4)	C1 < C3, C4
C20:4 n3	0.9 (0.6–1.3)	0.5 (0.1–0.7)	0.9 (0.4–1.0)	0.9 (0.8–1.1)	-
C20:5 n3 EPA	7.1 (5.1–10.1)	9.8 (8.3–11)	7.5 (3.4–10)	7.4 (6.3–8.5)	-
C22:0	0.1 (0–0.2)	0.1 (0.1–0.1)	0.2 (0.1–0.2)	0.1 (0–0.2)	-
c22:1 n11	9.2 (0.8–18.6)	4.8 (0.8–13.6)	0.3 (0.1–0.7)	8.8 (0.5–12.6)	C1, C4 > C3
C22:1 n9	0.6 (0.2–1.0)	0.3 (0.1–0.6)	0.2 (0.1–0.3)	0.4 (0.2–0.5)	C1 > C3
C22:2	0.1 (0–0.4)	0.3 (0.3–0.5)	0.3 (0.2–0.4)	0.4 (0.2–0.4)	C1 < C2, C3, C4
C22:3	0.1 (0–0.1)	0.1 (0–0.1)	0.1 (0–0.1)	0.0 (0–0.1)	-
C22:4	0.4 (0.2–0.7)	0.4 (0.2–0.5)	0.7 (0.4–1.0)	0.3 (0.2–0.3)	C1, C2, C4 < C3
c22:5 n3	0.8 (0.7–1.0)	0.9 (0.4–1.4)	0.6 (0.3–0.7)	0.8 (0.7–0.9)	-
C24:0	0.0 -	0.0 -	0.0 -	0.0 -	
C22:6 n3 DHA	14.7 (8–25.7)	22.0 (13.6–31.0)	22.4 (11.3–27.1)	16.1 (13.3–20)	C1 < C3
C24:1 n9	1.1 (0.8–1.6)	0.9 (0.5–1.4)	1.2 (0.6–1.6)	1.0 (0.8–1.2)	-
SFAs	24.2 (13.9–38.4)	29.3 (25.0–33.2)	35.9 (21.7–53.7)	32.3 (30.1–36.5)	C1 < C3
MUFAs	46.3 (24.8–61.9)	31.2 (21.0–39.7)	26.1 (19.5–36.6)	34.8 (26.9–39.7)	C1 > C2, C3
PUFAs	29.5 (22.0–43.5)	39.5 (33.2–47.1)	38.0 (19.9–44.6)	32.9 (28.4–36.5)	C1 < C2, C3
n-3	26.6 (16.5–37.9)	36.0 (30.1–43.9)	34.6 (18.0–40.7)	30.3 (26.2–34.1)	C1 < C2, C3

**Table 5:** Mass balance of the fractions obtained after processing with thermal treatment and enzymatic hydrolysis with commercial and endogenous enzymes. Values are shown as a percentage of the wet weight of raw material (g/100 g). T, E, PB, P, and C refer to thermal separation, endogenous enzymes, papain-bromelain, Protamex, and Corolase, respectively. The data are presented as mean values, and the minimum and maximum values are included in brackets. The variance among the different treatments (*p* < 0.05) is described with the symbols “<” and “>” that indicate if the value on the left is significantly higher or smaller than the value on the right, respectively.

Cruise	Lipid Content	Fractions	Thermal Separation	Endogenous Enzymes	Papain-Bromelain	Protamex	Corolase	*p* < 0.05
Cruise 1	9.9 (4.3–15.8)	Oil	9.6 (5.3–12.7)	7.4 (2.9–9.4)	7.2 (2.3–11.5)	5.9 (2.4–7.6)	6.3 (2.6–7.8)	
		Emulsion	-	7.9 (0–16.7)	13.4 (3.2–19.5)	17.9 (8.6–24.1)	17.5 (6.5–23.1)	
		Stickwater/Protein hydrolysate	34.9 (31.1–44.7)	17.9 (13.1–26.3)	28.2 (24.9–36.1)	27.6 (24.5–32.8)	27.5 (24.8–31.4)	T > E < PB, P, C
		Sludge/Sediments	55.5 (49.4–58.5)	66.8 (60.0–70.7)	51.2 (47.6–57.5)	48.6 (40.8–54.7)	48.7 (43.6–56.1)	T > E < PB, P, C
Cruise 2	2.3 (1.4–3.0)	Oil	1.3 (0–6.3)	-	-	-	-	
		Emulsion		-	-	-	-	
		Stickwater/Protein hydrolysate	42.7 (31.8–67.8)	-	-	-	-	
		Sludge/Sediments	56 (32.2–68.1)	-	-	-	-	
Cruise 3	4.9 (2.2–9.5)	Oil	1.3 (0–6.3)	-	-	-	-	
		Emulsion	-	-	-	-	-	
		Stickwater/Protein hydrolysate	39.6 (29.2–45.1)	-	-	-	-	
		Sludge/Sediments	59.1 (54.4–67.9)	-	-	-	-	
Cruise 4	9.8 (5.6–12.4)	Oil	6.8 (2.9–8.4)	6.6 (1.0–10.7)	9 (1.6–12.2)	7.7 (1.1–12.0)	-	
		Emulsion	-	-	-	-	-	
		Stickwater/Protein hydrolysate	42 (30.1–55.8)	36.4 (29–46.3)	39.2 (36.9–42.1)	38.1 (32.2–42.3)	-	
		Sludge/Sediments	51.2 (35.7–61.7)	57 (45.4–62.0)	51.8 (45.9–58.9)	54.2 (45.9–57.8)	-	

**Table 6:** Mass distribution of lipids and LC n-3 PUFAs calculated based on the total catch amount for each haul and the extrapolation of the total lipid content of the analyzed subsamples. The lipid content (%) and n-3% in lipids are presented as mean values ± SD.

Cruise	Haul	Total Catch (kg)	Lipids		n-3 in Lipids		n-3 in Raw Material
			%	kg	%	kg	%
Cruise 1	1	4000	15.8 ± 0.0	632.4	19.4 ± 0.1	122.4	3.1
	2	1000	11.0 ± 0.6	110.1	18.9 ± 0.2	20.9	2.1
	3	1000	13.7 ± 0.4	137.2	28.3 ± 0.2	38.8	3.9
	4	1000	12.0 ± 0.3	120.4	27.5 ± 0.1	33.0	3.3
	5	1000	12.2 ± 0.2	122.3	26.7 ± 0.1	32.6	3.3
	6	2000	6.2 ± 0.2	123.8	24.3 ± 0.1	30.1	1.5
	7	1000	4.3 ± 0.2	43.4	40.3 ± 0.1	17.5	1.7
	8	500	7.0 ± 0.0	35.15	26.3 ± 0.3	9.2	1.8
	9	100	5.4 ± 0.0	5.4	25.3 ± 0.2	1.4	1.4
	10	3000	11.1 ± 0.1	333.3	29.7 ± 0.0	98.9	3.3
	Average	1460	9.9 ± 0.2	166.3	26.6 ± 0.2	40.5	2.5
	Sum	14,600		1663		405	
Cruise 2	1	200	1.9 ± 0.1	3.8	44.0 ± 0.4	1.7	0.8
	3	350	1.4 ± 0.0	5.0	31.7 ± 0.1	1.6	0.5
	4	600	2.6 ± 0.1	15.8	38.1 ± 01	6.0	1.0
	5	500	3.0 ± 0.1	15.2	30.1 ± 0.1	4.6	0.9
	Average	515	2.3 ± 0.1	12.5	35.9 ± 0.2	3.5	0.8
	Sum	2060		50		14	
Cruise 3	1	300	4.3 ± 0.1	13.0	36.9 ± 0.0	4.8	1.6
	2	275	2.2 ± 0.0	6.1	39.5 ± 0.1	2.4	0.9
	3	100	3.2 ± 0.0	3.2	38.0 ± 0.5	1.2	1.2
	4	250	5.8 ± 0.0	14.6	37.8 ± 0.0	5.5	2.2
	5	300	3.7 ± 0.2	11.0	37.8 ± 0.3	4.2	1.4
	6	100	9.5 ± 0.1	9.5	30.3 ± 0.3	2.9	2.9
	7	300	2.5 ± 0.0	7.4	40.7 ± 0.0	3.0	1.0
	8	100	7.8 ± 0.2	7.8	34.7 ± 0.0	2.7	2.7
	Average	215.6	4.9 ± 0.1	9.1	37.0 ± 0.2	3.3	1.7
	Sum	1725		73		26	
Cruise 4	1	30.3	10.8 ± 0.2	3.3	34.1 ± 0.6	1.1	3.7
	2	90	12.4 ± 0.3	11.2	28.1 ± 0.1	3.1	3.5
	3	500	9.2 ± 0.1	45.9	33.0 ± 0.1	15.2	3.0
	4	500	8.3 ± 0.1	41.3	32.5 ± 0.1	13.4	2.7
	5	42	12.2 ± 0.1	5.1	26.2 ± 0.1	1.3	3.2
	6	110.1	9.8 ± 0.1	10.8	29.8 ± 0.1	3.2	2.9
	7	209.9	5.6 ± 0.1	11.8	28.7 ± 0.2	3.4	1.6
	Average	211.8	9.8 ± 0.1	18.5	30.3 ± 0.2	5.8	2.9
	Sum	1483		130		41	
	Total sum	19,868		1915		486	

## Data Availability

The original contributions presented in the study are included in the article, further inquiries can be directed to the corresponding author.
